# Relationship between weight-adjusted-waist index and erectile dysfunction in the United State: results from NHANES 2001-2004

**DOI:** 10.3389/fendo.2023.1128076

**Published:** 2023-04-25

**Authors:** Shangqi Cao, Xu Hu, Yanxiang Shao, Yaohui Wang, Yaxiong Tang, Shangqing Ren, Xiang Li

**Affiliations:** ^1^ Institute of Urology, Department of Urology, West China Hospital, West China Medical School, Sichuan University, Chengdu, China; ^2^ Robotic Minimally Invasive Surgery Center, Sichuan Academy of Medical Sciences & Sichuan Provincial People’s Hospital, Chengdu, China

**Keywords:** the weight-adjusted-waist index, erectile dysfunction, obesity, National Health and Nutrition Examination Survey, cross-sectional study

## Abstract

**Objective:**

The purpose of this study is to examine the association between a novel adiposity parameter, the weight-adjusted-waist index (WWI), and erectile dysfunction (ED).

**Methods:**

According to National Health and Nutrition Examination Survey (NHANES) 2001-2004, a total of 3884 participants were categorized as ED and non-ED individuals. WWI was calculated as waist circumference (WC, cm) divided by the square root of weight (kg). Weighted univariable and multivariable logistic regression models were conducted to assess the correlation between WWI and ED. Smooth curve fitting was utilized to examine the linear association. The receiver operating characteristic (ROC) curve and DeLong et al.’s test were applied to compare the area under curve (AUC) value and predictive power among WWI, body mass index (BMI), and WC for ED.

**Results:**

WWI was positively related to ED with the full adjustment [odds ratio (OR)=1.75, 95% confidence interval (95% CI): 1.32-2.32, p=0.002]. After converting WWI to a categorical variable by quartiles (Q1-Q4), compared to Q1 the highest WWI quartile was linked to an obviously increased likelihood of ED (OR=2.78, 95% CI: 1.39-5.59. p=0.010). Subgroup analysis revealed the stability of the independent positive relationship between WWI and ED. It was shown that WWI had a stronger prediction for ED (AUC=0.745) than BMI (AUC=0.528) and WC (AUC=0.609). Sensitivity analysis was performed to verify the significantly positive connection between WWI and stricter ED (OR=2.00, 95% CI: 1.36-2.94, p=0.003).

**Conclusion:**

An elevated WWI was related to higher risks of ED in the United State adults, and a stronger predictive power of WWI for ED was observed than BMI and WC.

## 1 Introduction

Erectile dysfunction (ED) is defined as the inability to obtain or maintain a sufficient erection for satisfactory intercourse (1). ED is a common disease that generally occurs in men older than the age of 40 years (2). According to the analysis from the Massachusetts Male Aging Study (3), the combined prevalence of mild, moderate, and complete ED was 52% in males from 40 to 70 years old. ED may be related to psychogenic factors or organic causes. In most patients, both reasons may lead to the occurrence of ED (4). Several risk factors and comorbidities are also closely associated with ED, such as obesity, lack of physical activity, smoking, alcoholism, diabetes mellitus, hypertension, dyslipidemia, and hypogonadism. In addition, ED was reported as a predictor for some illnesses including cardiovascular disease (CVD), coronary heart disease, and stroke (2, 5, 6). Obviously, ED brought a heavy health burden in increasingly aging men.

Obesity is a threat to the public health worldwide and is closely linked to many common diseases including but not limited to the following: hypertension, diabetes, CVD, cancers, psychiatric diseases, and sexual function (7, 8). Body mass index (BMI) is the most widely used parameter to evaluate obesity. However, the major deficiency of BMI is the failure to distinguish body fat from lean mass and central fat from peripheral fat (9). As a predictor of obesity, waist circumference (WC) has been proven to be related to visceral fat and abdominal obesity (10). However, it has been confirmed that there is a strong correlation between WC and BMI, and thus WC is limited as an independent measure of obesity (11). The weight-adjusted-waist index (WWI) is a novel index to evaluate obesity, proposed by Park et al. (12), and calculated using WC/weight^1/2^. Additionally, WWI presented a strong predictive power for cardiometabolic morbidity and mortality in their study (12). Furthermore, a previous study demonstrated that WWI was positively associated with fat mass but negatively connected with muscle mass (13).

Various evidence reveals that obesity is linked with increased odds of ED. Fillo et al. proved that males over the age of 40 with abdominal obesity had a higher risk of ED (14). Zhang et al. showed that subjects with BMI ≥ 30 kg/m^2^ had an elevated incidence of ED in China (15). However, the relationship between WWI and the prevalence of ED has not been explored. Therefore, within the National Health and Nutrition Examination Survey (NHANES) from 2001-2004, we investigated the correlation between WWI and ED and evaluated the predictive capability of WWI for ED compared to other indexes.

## 2 Materials and methods

### 2.1 Study description and population

The analyzed data were extracted from NHANES database, a major national program conducted by the Centers for Disease Control and Prevention’s National Center for Health Statistics (NCHS) with the purpose of evaluating the state of health and nourishment among the population of the United State. A unique feature of the survey is the harmonious combination of interviews and physical examinations. The survey interview comprises of demographic, socioeconomic, dietary, and health-related concerns. In addition, the examination elements encompass medical, dental, and physiological assessments, coupled with laboratory analyses executed by exceptionally skilled medical practitioners. Using a multistage probability design, NHANES collected a representative sample of noninstitutionalized civilian residents in the United States (16). The informed consent was signed by all participants in the NHANES study protocols that were approved by the Research Ethics Review Board of NCHS. More detailed information about the NHANES survey is available publicly at https://www.cdc.gov/nchs/nhanes/index.htm.

**Figure 1:**
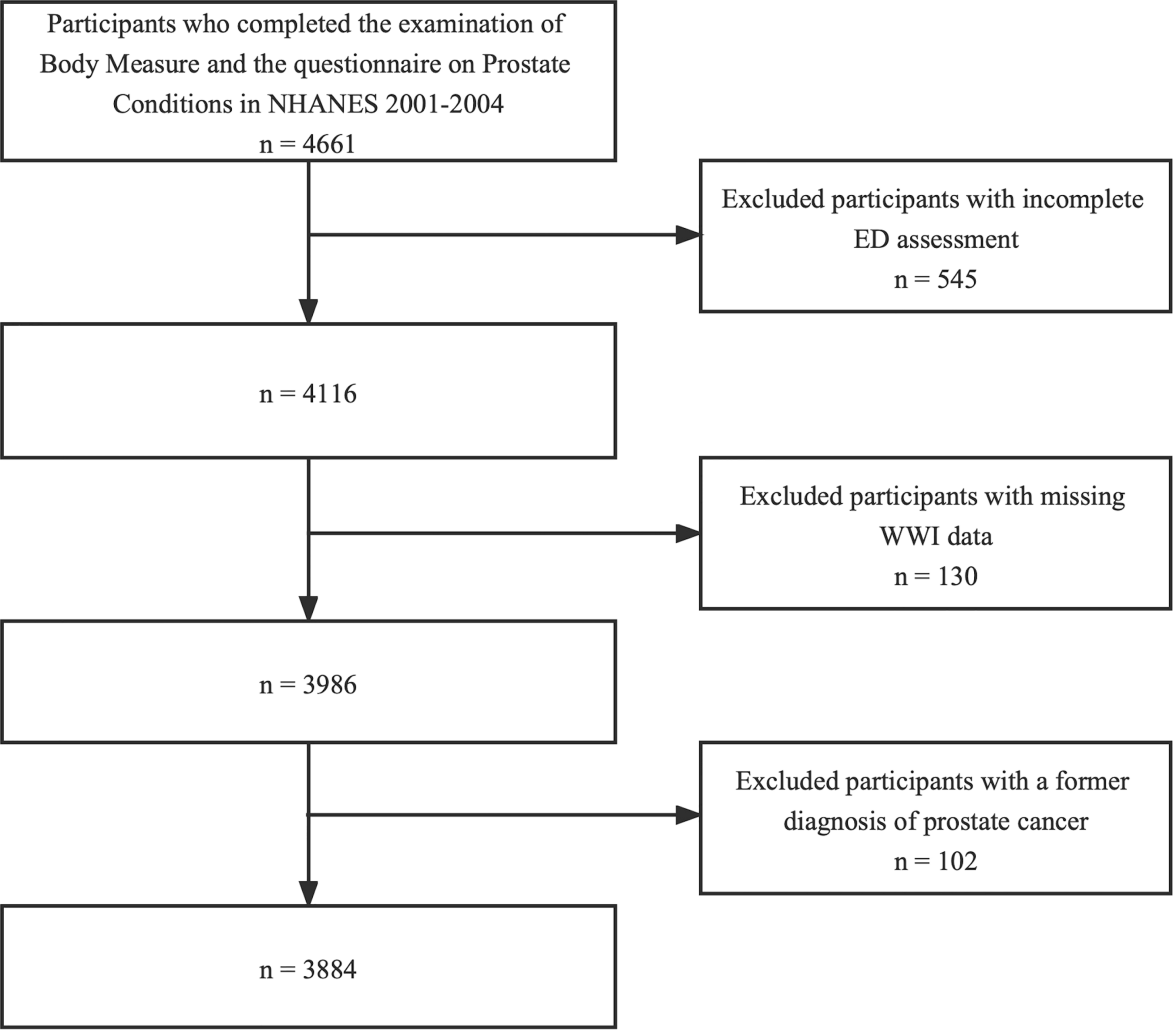
Flow chart of the sample selection process.

In this cross-sectional study, the data from two survey cycles (2001–2002, 2003–2004) were used because the ED information was available only in the period of 2001 to 2004. 4661 participants who completed the examination of “Body Measures” and the questionnaire on “Prostate Conditions” were enrolled at first. The participants with incomplete ED assessment (n=545), missing WWI data (n=130) and a former diagnosis of prostate cancer (n=102) were excluded. Ultimately, 3884 individuals were included in our study (**Figure 1**).

### 2.2 Measurement of WWI

The WWI is a novel adiposity indicator calculated as WC (cm) divided by the square root of weight (kg). The anthropometry assessments were conducted at the Mobile Examination Center (MEC) by proficient health technicians whose performance was monitored via direct observation, data reviews, and expert examiner evaluations. The “Body Measures” information on WC and weight are publicly available on the NHANES website. Detailed methods of anthropometrics are described at https://wwwn.cdc.gov/nchs/nhanes/continuousnhanes/manuals.aspx?BeginYear=2003. Weight was qualified by a Toledo digital scale and measured in pounds and converted to kilograms in the automated system. Study participants (SPs) should only be clothed in disposable paper gowns, underwear, and foam slippers for more accurate weight measurements. Additionally, SPs need to stand still in the center of the scale platform facing the recorder, hands at side, and looking straight ahead. WC is measured with a measuring tape. MEC health technicians will draw a horizontal line just above the uppermost lateral border of the right ilium. Subsequently, the right midaxillary line was drawn, and the measuring tape was placed horizontally at the point where the two lines intersected. And then technicians stand on the SP’s right side and encircle the measuring tape around the trunk in the horizontal plane. Generally, a higher WWI indicates more severe obesity. According to the WWI quartiles, survey individuals were divided into 4 groups(Q1-Q4) in further analysis. The WWI was set as both continuous and categorical variables in the analysis and was considered an exposure in our study.

### 2.3 Assessment of ED

The interview is conducted within a private room at the MEC, using an audio computer-assisted self-interview (ACASI) as the mode of administration. Self-assessment of ED designed as the outcome variable was conducted by a single question from the Massachusetts Male Aging Study (17): “Many men experience problems with sexual intercourse. How would you describe your ability to get and keep an erection adequate for satisfactory intercourse?” Response options included “always or almost always able”, “usually able”, “sometimes able”, and “never able”. In this analysis, ED was defined as participants who answered “sometimes able” or “never able” to keep an erection. Survey individuals who answered “always or almost always able” or “usually able” to keep an erection were classified as not having ED. In a sensitivity analysis, we redefined ED stringently as participants who answered “never able” to keep an erection (18).

### 2.4 Covariates of interest

In the current study, covariates of interest included age, race, education level, marital status, the family poverty income ratio (PIR), BMI, physical activity (vigorous/moderate), smoking status, alcohol intaking, diabetes, hypertension, high cholesterol, and CVD. Physical activity was classified as vigorous activity (yes or no) and moderate activity (yes or no). Survey individuals smoking at least 100 cigarettes in their lives and smoking when taking the questionnaire were designed as current smokers. Former smokers are defined as someone who smoked at least 100 cigarettes throughout their lifetime and did not smoke at the time of the questionnaire. In addition, males who smoked less than 100 cigarettes during their lifetime were defined as nonsmokers. Participants who answered “no” to drinking at least 12 alcohol drinks in their entire life and in any one year were designed as nondrinkers. Study males who answered “yes” to drinking 12 alcohol drinks in their entire life or in any one year but consumed no drinks in the past 12 months were set as former drinkers. Study individuals were current drinkers if they answered “yes” to drinking 12 alcohol drinks in their entire life or in any one year and they drank at least one alcohol drink in the past 12 months (19). If doctors had told participants that they had diabetes or their fasting plasma glucose was ≥126mg/dL, they were considered to have diabetes. Survey individuals with hypertension were designated as those who had a prior diagnosis of hypertension, or were taking medication for hypertension, or had a systolic or diastolic blood pressure greater than or equal to 140 mmHg or 90 mmHg respectively. Participants who were told by doctors that their blood cholesterol level was high, or were taking a prescription for hypercholesterolemia, or had a total cholesterol value ≥ 240 mg/dL were considered to have high cholesterol. CVD refers to participants who have previously been diagnosed with congestive heart failure, or coronary heart disease, or angina, or heart attack.

### 2.5 Statistical analysis

In the present study, the multistage design of the NHANES was taken into consideration in all statistical analyses by selecting appropriate sampling weights, strata, and primary sampling units. In the baseline characteristics table, continuous variables were expressed as the weighted mean and standard error (SE), and categorical variables were shown as weighted proportions. A survey-weighted linear regression for continuous variables and a survey-weighted Chi-square test for categorical variables were utilized to evaluate the differences between participants with or without ED. The analysis of univariable and multivariable logistic regressions was performed to examine the relationship between WWI level and the prevalence of ED. There were 3 models applied in the study: Model 1 was unadjusted; Model 2 was adjusted for age, race, and BMI; Model 3 was adjusted for age, race, BMI, education level, marital status, the family PIR, smoking status, alcohol intaking, vigorous activity, moderate activity, diabetes, hypertension, high cholesterol, and CVD.

Subgroup analyses were conducted to investigate the correlation between WWI and ED in different subgroups. For sensitivity analysis, we classified participants who answered “never able” to maintain an erection as having stricter ED. Multivariable logistic regression models also were utilized in the sensitivity analysis. The smooth curve fitting and generalized addition model were utilized to examine whether the independent variable was divided into different intervals, which meant whether the independent variable was non-linearly related to ED. The receiver operating characteristic (ROC) curve and the area under curve (AUC) were presented to assess the predictive power of WWI, BMI, and WC for ED. The comparison of AUC between WWI and other indicators was verified by Delong et al.’s test (20). A two-sided p-value of < 0.05 was considered statistically significant. All statistical analyses were conducted using EmpowerStats (http://www.empowerstats.com, X&Y Solutions, Inc.) and statistical software packages R (http://www.R-project.org; The R Foundation).

## 3 Results

### 3.1 Participant characteristics

A total of 3884 survey individuals aged from 20 to 85 years were enrolled in our study. 1056 of 3884 (27.19%) individuals had a history of ED. WWI was significantly elevated in the ED participants compared to that in the participants without ED [11.22 (0.03) cm/√kg vs 10.59 (0.02) cm/√kg, p<0.001]. Males with ED may be older, less educated, married or living with partner, have lower socio-economic status, higher BMI level, less physical activity, be former smokers, and have diabetes, hypertension, high cholesterol, and CVD (all p<0.05). The detailed demographic data of all survey individuals are demonstrated in **Table 1**.

**Table 1:** Baseline characteristics of participants by a history of erectile dysfunction, weighted.

Characteristic	History of erectile dysfunction		P-value
	No (n=2828)	Yes (n=1056)	
**Age (year), mean (SE)**	41.07 (0.32)	60.62 (0.48)	<0.001
**WII (cm/√kg), mean (SE)**	10.59 (0.02)	11.22 (0.03)	<0.001
Age (%)			<0.001
<50	74.04	20.02	
≥50	25.96	79.98	
BMI (%)			0.002
<25	31.12	24.87	
≥25 and <30	41.20	40.33	
≥30	27.68	34.81	
Race (%)			0.450
Mexican American	7.88	7.38	
Other Hispanic	4.14	5.34	
Non-Hispanic White	73.63	75.32	
Non-Hispanic Black	10.05	8.55	
Other race	4.30	3.41	
Education level (%)			<0.001
Less than high school	13.98	29.25	
High school or GED	27.89	23.11	
Above high school	58.13	47.64	
Marital status (%)			<0.001
Married or living with partner	67.76	76.06	
Living alone	32.24	23.94	
Family PIR (%)			<0.001
≤ 1.3	16.19	19.35	
> 1.3 and ≤ 3.5	33.77	41.87	
> 3.5	50.04	38.78	
Smoking status (%)			<0.001
Current smokers	29.54	23.84	
Former smokers	24.93	46.74	
Nonsmokers	45.53	29.42	
Alcohol intaking (%)			0.038
Current drinkers	79.67	64.30	
Former drinkers	13.36	29.44	
Nondrinkers	6.97	6.25	
Vigorous activity (%)			<0.001
No	55.58	81.31	
Yes	44.42	18.69	
Moderate activity (%)			<0.001
No	42.06	52.63	
Yes	57.94	47.37	
Diabetes (%)			<0.001
No	95.07	73.64	
Yes	4.93	26.36	
Hypertension (%)			<0.001
No	71.23	40.20	
Yes	28.77	59.80	
High cholesterol (%)			<0.001
No	66.32	48.43	
Yes	33.68	51.57	
CVD (%)			<0.001
No	95.51	76.50	
Yes	4.49	23.50	

SE, standard error; WWI, weight-adjusted-waist index; BMI, body mass index; GED, general educational development; Family PIR, family poverty income ratio; CVD, cardiovascular disease.

### 3.2 The association between WWI and ED

**Figure 2:**
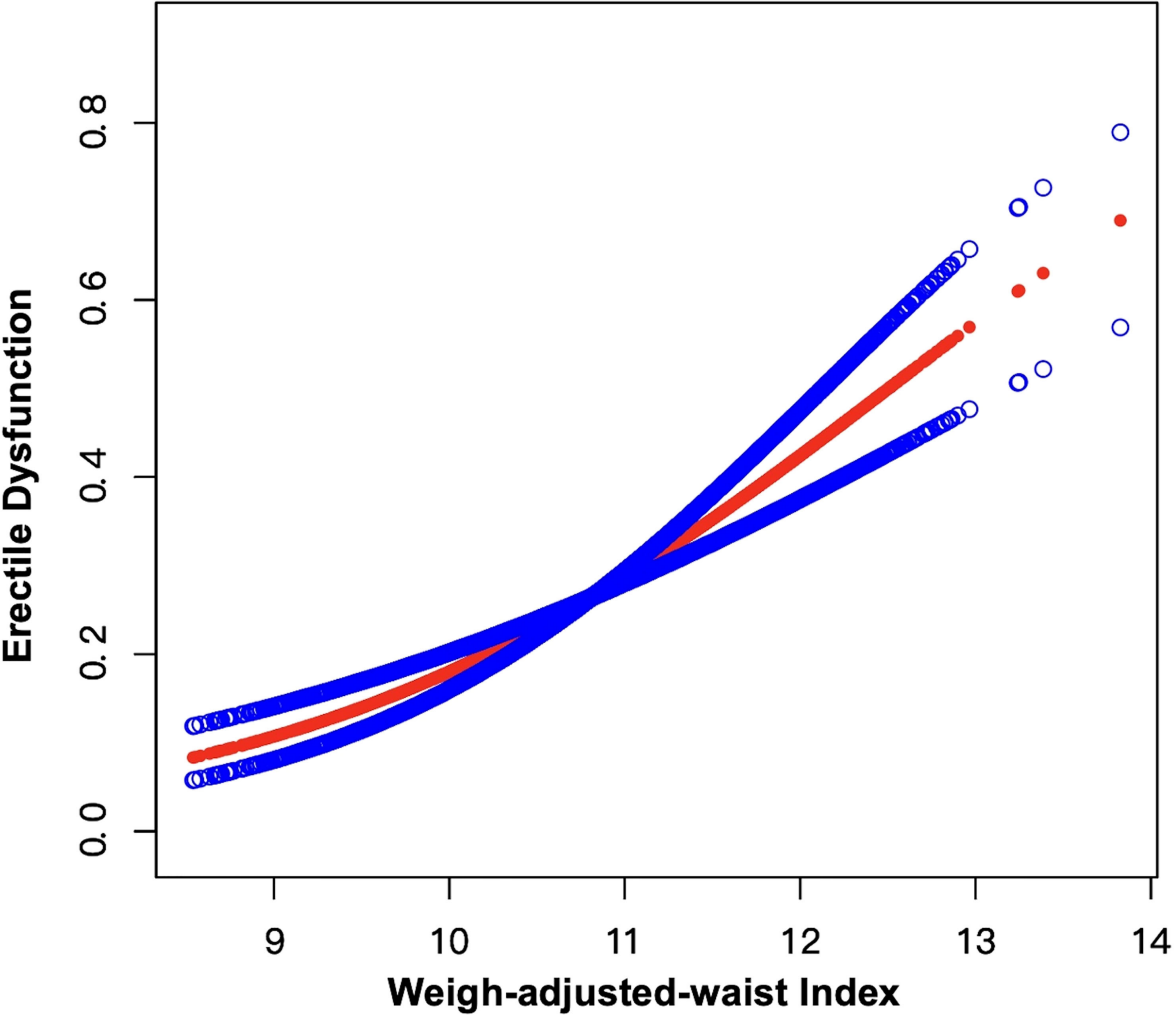
Smooth curve fitting for WWI and ED. The area between the upper and lower blue dotted lines is on behalf of 95% CI. The red dotted line indicates that the positive linear association between WWI and ED is proven by generalized additive model.

Weighted univariable logistic regression was conducted to assess the association of WWI and all chosen covariates with ED. Detailed information was shown in **Table 2**. We used the weighted multivariable logistic regression to examine the relationship between WWI and ED in crude, minimally adjusted, as well as fully adjusted models, and found a significant positive connection between WWI and ED in all models, which was demonstrated in **Table 3**. In the fully adjusted model (Model 3), each unit increase in WWI was related to a 75% greater risk of ED [odds ratio (OR)=1.75, 95% confidence interval (95% CI): 1.32-2.32, p=0.002]. Additionally, the continuous WWI variable was transformed into a categorical variable by WWI quartiles and the positive relationship between four WWI intervals and ED maintained statistically significant. In model 3, survey individuals in the highest quartile (Q4) had 178% greater risks of ED compared to those in the lowest quartile (Q1) (OR=2.78, 95% CI:1.39-5.59, p for trend=0.004). Furthermore, an analysis of smooth curves fitting also revealed a positive linear correlation between WWI and ED (**Figure 2**).

**Table 2:** Weighted univariable logistic regression analysis for the relationship of WWI and all chosen covariates with ED.

	OR (95%CI)	P-value
**WWI**	3.52 (2.95,4.20)	<0.001
WWI quartiles		
Q1	**Reference**	
Q2	2.33 (1.70, 3.20)	<0.001
Q3	4.90 (3.36, 7.16)	<0.001
Q4	11.79 (7.97, 17.43)	<0.001
Age		
<50	**Reference**	
≥50	11.40 (9.51, 13.66)	<0.001
BMI		
<25	**Reference**	
≥25 and <30	1.23 (0.99, 1.51)	0.070
≥30	1.57 (1.21, 2.05)	0.002
Race		
Mexican American	**Reference**	
Other Hispanic	1.38 (0.72, 2.62)	0.339
Non-Hispanic White	1.09 (0.83, 1.44)	0.536
Non-Hispanic Black	0.91 (0.67, 1.22)	0.531
Other race	0.85 (0.45, 1.61)	0.616
Education level		
Less than high school	**Reference**	
High school or GED	0.40 (0.31, 0.51)	<0.001
Above high school	0.39 (0.32, 0.47)	<0.001
Marital status		
Married or living with partner	**Reference**	
Living alone	0.66 (0.54, 0.81)	<0.001
Family PIR		
≤ 1.3	**Reference**	
>1.3 and ≤ 3.5	1.04 (0.82, 1.31)	0.760
>3.5	0.65 (0.53, 0.79)	<0.001
Smoking status		
Nonsmokers	**Reference**	
Former smokers	2.90 (2.45, 3.44)	<0.001
Current smokers	1.25 (0.98, 1.60)	0.086
Alcohol intaking		
Nondrinkers	**Reference**	
Former drinkers	2.46 (1.50, 4.03)	0.001
Current drinkers	0.90 (0.55, 1.48)	0.681
Vigorous activity		
No	**Reference**	
Yes	0.29 (0.22, 0.37)	<0.001
Moderate activity		
No	**Reference**	
Yes	0.65 (0.54, 0.79)	0.001
Diabetes		
No	**Reference**	
Yes	6.90 (5.16, 9.22)	<0.001
Hypertension		
No	**Reference**	
Yes	3.68 (3.16, 4.29)	<0.001
High cholesterol		
No	**Reference**	
Yes	2.10 (1.76, 2.50)	<0.001
CVD		
No	**Reference**	
Yes	6.53 (5.16, 8.26)	<0.001

OR, odds ratio; 95% CI, 95% confidence interval; Q1-Q4, quartile 1-quartile 4. WWI, weight-adjusted-waist index; BMI, body mass index; GED, general educational development; Family PIR, family poverty income ratio; CVD, cardiovascular disease.

**Table 3:** Weighted multivariable logistic regression for the association between weight-adjusted-waist index and erectile dysfunction.

	OR (95% CI), P-value		
	Model 1	Model 2	Model 3
**Continuous**	3.53 (2.95, 4.20), <0.001	2.47 (2.03, 3.01), <0.001	1.75 (1.32, 2.32), 0.002
Categories			
**Q1**	**Reference**	**Reference**	**Reference**
Q2	2.33 (1.70, 3.20), <0.001	1.75 (1.27, 2.42), 0.003	1.54 (0.92, 2.57), 0.069
Q3	4.90 (3.36, 7.16), <0.001	2.82 (1.97, 4.04), <0.001	2.12 (1.15, 3.93), 0.019
Q4	11.79 (7.97, 17.43), <0.001	5.25 (3.38, 8.17), <0.001	2.78 (1.39, 5.59), 0.010
**P for trend**	**<0.001**	**<0.001**	**0.004**

OR, odds ratio; 95% CI, 95% confidence interval; Q1-Q4, quartile 1-quartile 4.

Model 1: Unadjusted; Model 2: Adjusted for age, race, and body mass index; Model 3: Adjusted for age, race, body mass index, education level, marital status, the family poverty income ratio, smoking status, alcohol intaking, vigorous activity, moderate activity, diabetes, hypertension, high cholesterol, and cardiovascular disease.

### 3.3 Subgroup analysis

**Figure 3:**
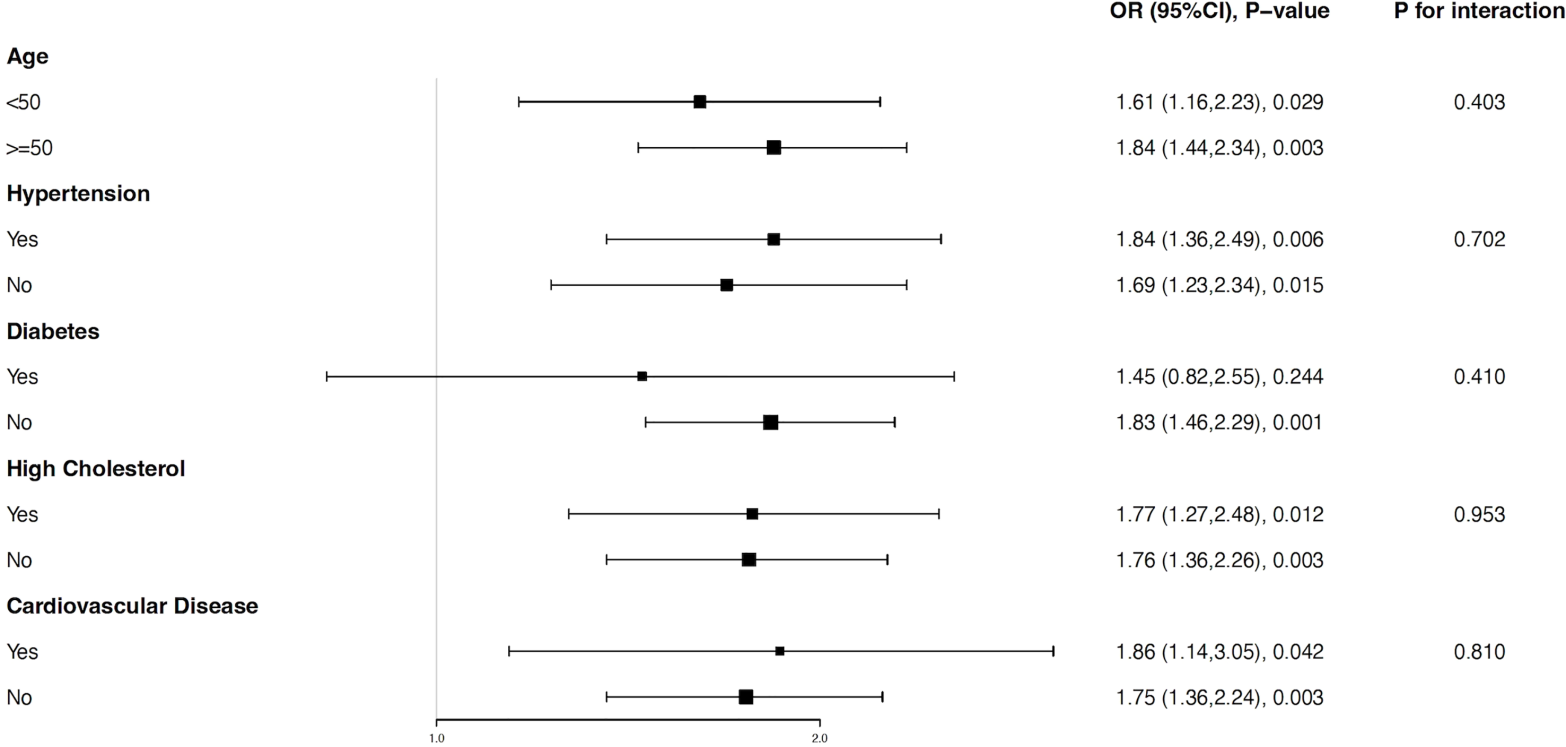
Subgroup analysis for the relationship between weight-adjusted-waist index and erectile dysfunction. All factors including age, a history of hypertension, diabetes, high cholesterol, and CVD had no impact on the independent positive association between WWI and ED. All subgroups were adjusted for age, race, body mass index, education level, marital status, the family poverty income ratio, smoking status, alcohol intaking, vigorous activity, moderate activity, diabetes, hypertension, high cholesterol, and cardiovascular disease, except the stratification factor itself.

Subgroup analyses were performed to explore whether the WWI-ED association was steady in different stratifications. As shown in **Figure 3**, all subgroups including age, a history of hypertension, diabetes, high cholesterol, and CVD had no impact on the independent positive WWI-ED correlation (all p for interaction > 0.05). In some stratifications, the positive relationship was statistically stably significant. For instance, each unit of increased WWI was related to a 61% higher likelihood of ED for participants younger than 50 years old (OR=1.61, 95% CI: 1.16-2.23, p=0.029) and an 84% higher risk of ED for those aged 50 and older (OR=1.84, 95% CI: 1.44-2.32, p=0.003). Detailed analytical results were presented in **Figure 3**.

### 3.4 WWI had a stronger predictive ability for ED than BMI and WC

**Figure 4:**
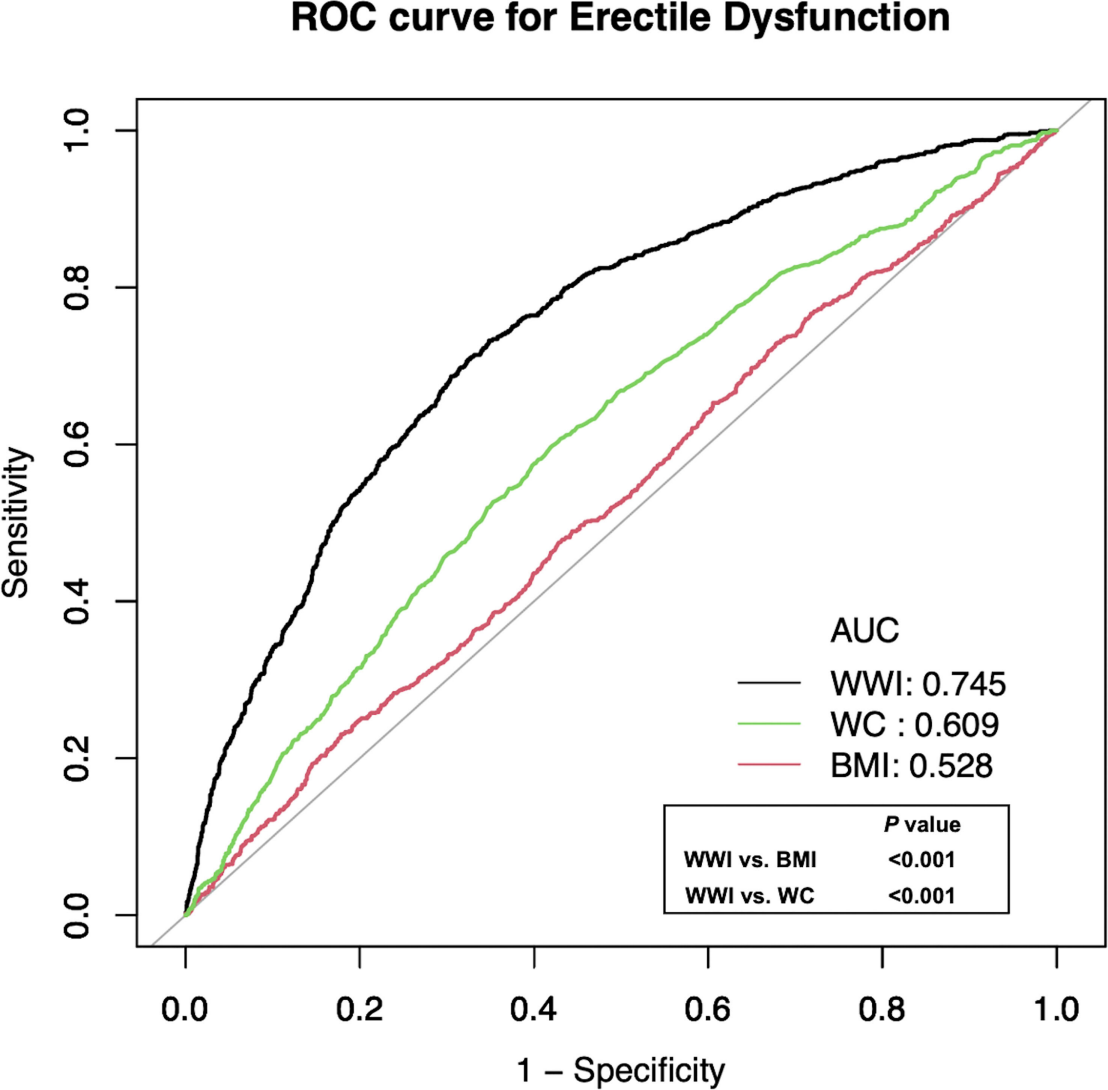
Receiver operating characteristic (ROC) curve analysis for predicting ED. Delong et al.’s method for comparison of area under curve (AUC) value between WWI and BMI, WC.

In **Table 4**, the AUC values (95% CI) of the anthropometric indicators were shown: WWI: 0.745 (0.728-0.762), BMI: 0.528 (0.507-0.549), WC: 0.609 (0.589-0.629). Of the three parameters, it has been shown that the highest AUC was WWI. **Table 4** also included several details of all the adiposity indexes including cutoff value, sensitivity, and specificity. For instance, the optimum cutoff value of WWI predicting ED was 10.920 (specificity: 0.652, sensitivity: 0.732). In **Figure 4**, ROC curves were conducted to assess the prediction of adiposity indexes for ED. In addition, the comparisons between WWI and other parameters demonstrated that the differences in AUC values between WWI and BMI, WC were statistically significant (both p<0.001), indicating that WWI may be a better predictive index of ED than BMI and WC.

**Table 4:** The adiposity indicators for predicting ED.

Test	AUC	95%CI low	95%CI upp	Cutoff value	Specificity	Sensitivity
WWI	0.745	0.728	0.762	10.920	0.652	0.732
BMI	0.528	0.507	0.549	31.775	0.822	0.231
WC	0.609	0.589	0.629	99.950	0.581	0.597

AUC, area under curve; 95% CI, 95% confidence interval.

### 3.5 Sensitivity analysis

**Figure 5:**
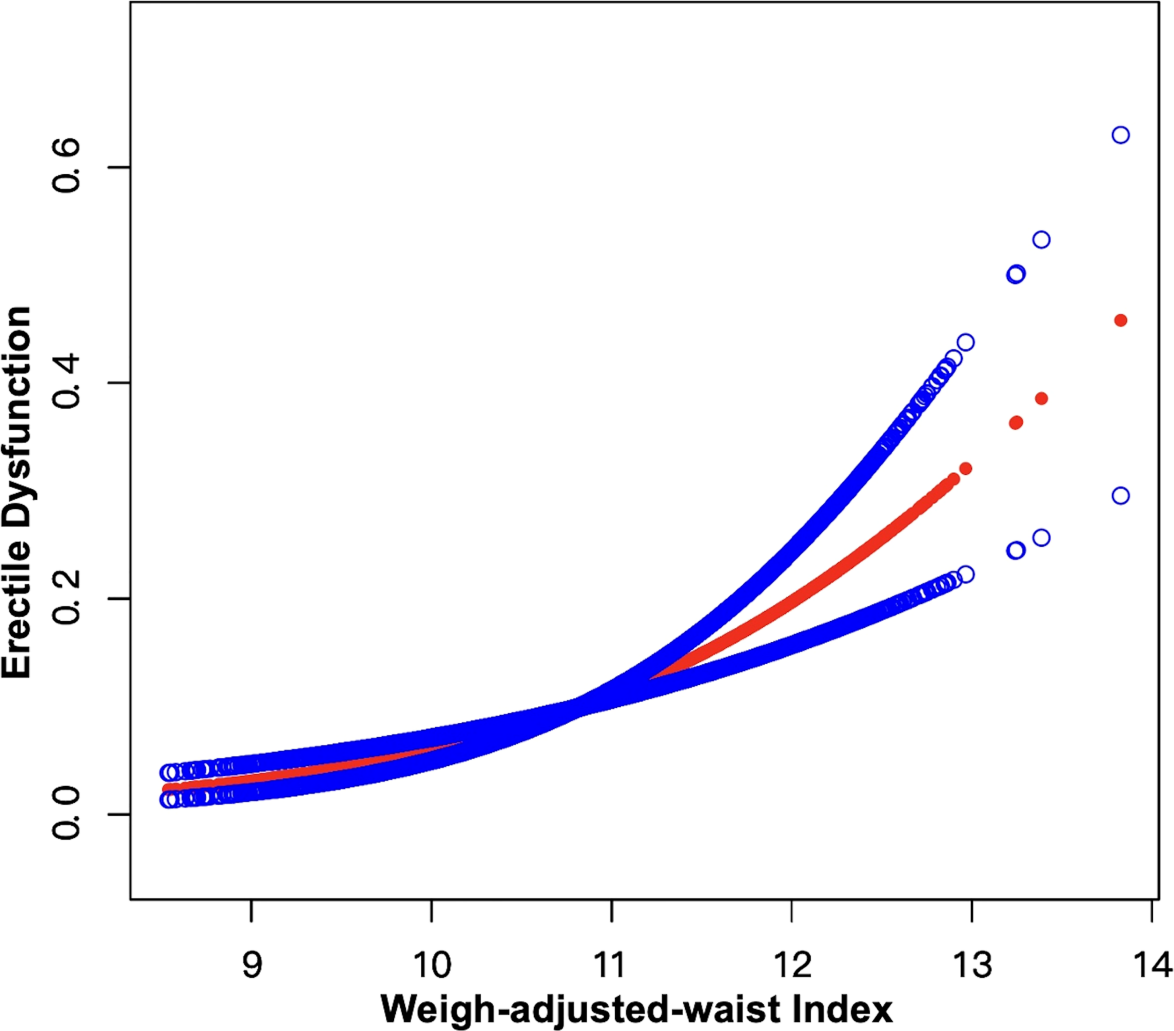
Smooth curve fitting for WWI and stricter ED. The area between the upper and lower blue dotted lines is on behalf of 95% CI. The red dotted line indicates that the positive linear association between WWI and stricter ED is proven by generalized additive model.

We designed the participants who answered “never able” to keep an erection as stricter ED individuals for sensitivity analyses. As shown in **Table 5**, positive associations were observed in all models. In Model 3, one additional unit of WWI was correlated with a 100% increased risk of ED (OR=2.00, 95% CI: 1.36-2.94, p=0.003). In addition, we explored the linear association between WWI and stricter ED using a smooth curve fitting and generalized addition model (**Figure 5**), and WWI was still linearly positively related to stricter ED.

**Table 5:** Sensitivity analysis for the association between WWI with stricter ED.

	OR (95% CI), P-value		
	Model 1	Model 2	Model 3
WWI	3.97 (3.34, 4.73), <0.001	2.82 (2.19, 3.64), <0.001	2.00 (1.36, 2.94), 0.003

OR, odds ratio; 95% CI, 95% confidence interval. Q1-Q4, quartile 1-quartile 4.

Model 1: Unadjusted; Model 2: Adjusted for age, race, and body mass index; Model 3: Adjusted for age, race, body mass index, education level, marital status, the family poverty income ratio, smoking status, alcohol intaking, vigorous activity, moderate activity, diabetes, hypertension, high cholesterol, and cardiovascular disease.

## 4 Discussion

In this cross-sectional study, we explored the connection between WWI and ED in noninstitutionalized U.S. residents and discovered that an elevated WWI was strongly linked with a higher likelihood of ED. The positive connection still existed when WWI was transformed into a categorical variable by quartiles (Q1-Q4), Subgroup analyses revealed that all stratification variables had no effect on the stability of the relationship between WWI and ED and the positive correlation persisted. Moreover, the ROC curve and Delong et al.’s method were utilized to explore and compare the predictive efficiency for ED between WWI and BMI, WC, respectively, and found that WWI had a stronger predictive power for ED compared to BMI and WC. Lastly, we used a stricter definition of ED for sensitivity analysis and found that the impact of WWI on ED was enhanced. As far as we know, this is the first study to focus on the correlation between WWI and ED.

The association between obesity and ED has been supported by numerous studies. Bacon et al. conducted a 14-year prospective study for investigating the risk factors of ED and found obesity was positively associated with ED (21). Riedner et al. demonstrated that several anthropometric parameters evaluating central obesity were linked to the likelihood of ED in men older than 60 years old (22). There are many factors and mechanisms involved in the regulation of obesity and ED. For instance, the alteration of sex hormone metabolism caused by obesity is one of the crucial factors affecting ED. Corona et al. showed that with adjustment of comorbidities, the lower level of androgen is a characteristic feature of obesity in men with ED (23). Besides a decrease in testosterone, obese males present a decrease in the testosterone-to-estradiol ratio and an elevated level of estrogen, including estrone and estradiol, which play a significant role in the development of ED by exerting a regulatory effect on the physiological processes involved in sexual performance (24). A study in rabbits indicated that ED induced by the high-fat diet was more closely related to elevated estradiol levels than to low testosterone levels (25). Insulin resistance related to obesity may have an impact on endothelial dysfunction which is a potential mechanism of ED formation (26). Physical activities also play important roles in the formation of ED. A meta-analysis proposed that physical activity and exercise could yield improvement in instances of ED. Notably, the greater benefit seemed to be derived from engaging in aerobic exercise with moderate-to-vigorous levels of intensity (27). Morelli et al. demonstrated that physical exercise could reverse some characteristics of high-fat diet rabbits with metabolic syndrome (MetS). For example, physical exercise had the potential to improve erectile function and increase luteinizing hormone and testosterone concentration (28). Certain comorbidities related to obesity have also been shown to have an impact on the development of ED. Nonalcoholic fatty liver disease (NAFLD) is closely associated with MetS and is recognized as the hepatic manifestation of MetS. NAFLD has been shown to be significantly linked to ED in a prospective pilot study (29). Vignozzi et al. showed that the emergence of MetS-induced nonalcoholic steatohepatitis in high-fat diet rabbits was thought to actively participate in the pathogenesis of ED via an increase of TNFα (30). Moreover, obesity is also closely related to female sexual dysfunction. A cross-sectional study indicated that obesity was associated with an elevated likelihood of dysfunction in desire and arousal and reduced sexual satisfaction in postmenopausal females (31). Werlinger et al. investigated that weight loss promoted the overall perception of sexual function in females (32). Therefore, it is necessary to evaluate the degree of obesity to predict the odds of ED.

The WWI is regarded as a new obesity indicator and has been shown to be related to various factors, especially CVD. Ding et al. demonstrated that there was a relationship between higher WWI levels (≥ 11.2 cm/√kg) and increased odds of all-cause (HR=1.36, 95% CI: 1.17-1.58) and cardiovascular mortality (HR=1.43, 95% CI: 1.15-1.77) in southern China (33). A rural Chinese cohort study showed a statistically significant association between the highest WWI level (≥ 10.91 cm/√kg) and a higher incidence of hypertension (OR=1.50, 95%CI: 1.24-1.82) (34). It has been confirmed that ED and CVD have many common risk factors including but not limited to hypertension, diabetes, obesity, and smoking. In addition, some pathophysiological mechanisms, such as endothelial dysfunction, inflammation, aging, and low plasma testosterone levels, have an impact on both ED and CVD (35, 36). It has been shown that ED is an independent predictor of CVD and provides opportunities for prevention and treatment (37). Interestingly, cardiovascular events have been studied to be related to female sexual dysfunction (38). Maseroli et al. demonstrated that clitoral vascular resistance evaluated by color Doppler ultrasound was positively associated with decreased sexual arousal (39). Therefore, the relationship between CVD and sexual dysfunction is widely confirmed. Furthermore, there was strong evidence that WWI was significantly positively related to abdominal fat (13, 40). Riedner et al. revealed that central obesity evaluated by several indicators was highly associated with ED in older males (22). Janiszewski et al. demonstrated that abdominal obesity assessed by a high WC was independently linked with increased odds of ED (41). Moreover, MetS is a metabolic disorder and a cluster of risk factors of CVD, such as visceral adiposity, insulin resistance, inflammation, and endothelial dysfunction (42). Meanwhile, MetS has been shown to be correlated with ED. Heidler et al. explored the correlation between MetS and ED in a total of 2371 men and discovered that only in males ≥ 50 years old, a higher prevalence of MetS was related significantly to a greater risk of ED, and subjects with severe ED rose by 48% (43). García-Cruz et al. showed that survey individuals with MetS had significantly lower scores on International Index of Erectile Function-5 (IIEF-5) (p<0.001). Additionally, the incidence of moderate-to-severe ED was higher among participants with MetS than those without MetS (p<0.001) (44). WWI as an indicator of abdominal obesity may be linked to MetS. Therefore, it is reasonable to speculate that WWI may have a positive correlation with ED prevalence.

As we all know, BMI is one of the most commonly used indicators for assessing obesity. However, a main limitation of BMI is that it neither differentiates body fat from lean mass nor evaluates the distribution of body mass. For instance, an individual with more muscle mass and less fat mass may be mistaken for having a high BMI, while a subject with increased fat mass and little lean mass may be considered to have a normal BMI (9, 45). WC is always utilized to assess central obesity. Pizzol et al. demonstrated that overweight (BMI: 25–29.9 kg/m^2^) individuals had higher odds of ED than individuals with normal weight (OR = 1.31, 95% CI: 1.13–1.51). In addition, the incidence of ED was higher in participants with obesity (BMI ≥ 30kg/m^2^) than in normal-weight individuals (OR = 1.60, 95% CI: 1.29–1.98). Moreover, the association between higher ED prevalence and an elevated WC was proved. Therefore, higher BMI and WC have been confirmed to evaluate the increased risk of ED (46). Yassin et al. showed that WC was significantly negatively associated with scores on IIEF-5, which indicated higher WC values were related to an increased ED prevalence (47). In addition, the visceral adiposity index (VAI) is a novel obesity indicator that has higher sensitivity and specificity than some classical parameters, such as BMI and WC (48). Some studies have explored the correlation between visceral obesity evaluated by VAI with ED. Dursun et al. demonstrated that the mean VAI value of ED males was 5.18 ± 2.50 and of non-ED males was 3.47 ± 1.76. Compared with non-ED individuals, VAI levels were statistically higher in the ED subgroup (p < 0.001) (49). Bolat et al. conducted a multivariable logistic regression analysis and discovered that each additional unit of VAI was linked to 30% greater odds of ED (95% CI: 1.08-1.58). Therefore, VAI can be regarded as an independent predictor of ED (50). There is more and more evidence revealing that many indicators evaluating obesity are closely associated with ED. However, obesity paradox is a controversial topic on cardiometabolic risk. There is a paradoxical association between higher BMI and lower risk of cardiovascular events in individuals with CVD (51). More research is needed to confirm whether the obesity paradox exists in the ED population. Moreover, Park et al. indicated that WWI may be a better index representing WC and is weakly correlated with BMI (12). It is worth mentioning that some novel anthropometric indexes such as VAI are calculated by relatively complex mathematical formulas, resulting in the inconvenience of use, while WWI is calculated by a relatively simple formula for the convenience of the routine examination. In our study, we concluded that WWI has a stronger relationship with ED than BMI and WC. Therefore, WWI as an anthropometric index has good results in independently predicting ED prevalence and is expected to be further studied for predicting the risk of other diseases.

There are several strengths in our study. We used a large sample of data from NHANES which is representative of the United States population and took full consideration of sample design and weighting. In addition, we adjusted related covariates in the multivariable logistic regression analyses for exploring the independent effect of WWI on ED. Furthermore, we conducted subgroup analyses for examining the stability of impact. Moreover, the sensitivity analysis is used to evaluate the association between the stricter ED and WWI. However, there are some limitations in our study. For instance, the current study is a cross-sectional study, which cannot explore causality. Additionally, a history of ED is self-reported by survey participants, possibly resulting in a smaller number of collected ED males than the true number of ED men. Furthermore, WWI as a new obesity index has not been widely used to evaluate obesity or central obesity in daily and clinical conditions compared to BMI and WC. More research needs to figure out the advantages and disadvantages of WWI in different clinical applications. Lastly, NHANES database only represents the population from the United State. Whether the relationship between WWI and ED exists in other national or regional populations needs to be verified by more studies.

## 5 Conclusion

This study showed that a higher WWI was related to an increased risk of ED prevalence. Additionally, compared to BMI and WC a stronger predictive capability for ED was observed in WWI. WWI may be a better adiposity indicator for evaluating ED. However, our results are needed to be verified by more studies.

## Data availability statement

The original contributions presented in the study are included in the article/supplementary material. Further inquiries can be directed to the corresponding author.

## Ethics statement

The studies involving human participants were reviewed and approved by the Research Ethics Review Board of the NCHS. The patients/participants provided their written informed consent to participate in this study.

## Author contributions

SC, XH, and YS performed the data analysis. SC, YW, YT and SR collected the data. SC, XH and XL designed this study and drafted and reviewed the manuscript. All authors contributed to the article and approved the submitted version.

## References

[1] Yafi FA Jenkins L Albersen M Corona G Isidori AM Goldfarb S. Erectile dysfunction. Nat Rev Dis Primers (2016) 2:16003. doi: 10.1038/nrdp.2016.3 27188339 PMC5027992

[2] Shamloul R Ghanem H. Erectile dysfunction. Lancet (2013) 381(9861):153–65. doi: 10.1016/s0140-6736(12)60520-0 23040455

[3] Feldman HA Goldstein I Hatzichristou DG Krane RJ McKinlay JB. Impotence and its medical and psychosocial correlates: results of the Massachusetts Male aging study. J Urol (1994) 151(1):54–61. doi: 10.1016/s0022-5347(17)34871-1 8254833

[4] Muneer A Kalsi J Nazareth I Arya M. Erectile dysfunction. Bmj (2014) 348:g129. doi: 10.1136/bmj.g129 24468580

[5] Rosen RC Wing R Schneider S Gendrano N 3rd. Epidemiology of erectile dysfunction: the role of medical comorbidities and lifestyle factors. Urol Clin North Am (2005) 32(4):403–17. doi: 10.1016/j.ucl.2005.08.004 16291033

[6] Dong JY Zhang YH Qin LQ. Erectile dysfunction and risk of cardiovascular disease: meta-analysis of prospective cohort studies. J Am Coll Cardiol (2011) 58(13):1378–85. doi: 10.1016/j.jacc.2011.06.024 21920268

[7] Larsen SH Wagner G Heitmann BL. Sexual function and obesity. Int J Obes (Lond) (2007) 31(8):1189–98. doi: 10.1038/sj.ijo.0803604 17372616

[8] De Lorenzo A Gratteri S Gualtieri P Cammarano A Bertucci P Di Renzo L. Why primary obesity is a disease? J Transl Med (2019) 17(1):169. doi: 10.1186/s12967-019-1919-y 31118060 PMC6530037

[9] Oliveros E Somers VK Sochor O Goel K Lopez-Jimenez F. The concept of normal weight obesity. Prog Cardiovasc Dis (2014) 56(4):426–33. doi: 10.1016/j.pcad.2013.10.003 24438734

[10] Ness-Abramof R Apovian CM. Waist circumference measurement in clinical practice. Nutr Clin Pract (2008) 23(4):397–404. doi: 10.1177/0884533608321700 18682591

[11] Mahmoud I Al-Wandi AS Gharaibeh SS Mohamed SA. Concordances and correlations between anthropometric indices of obesity: a systematic review. Public Health (2021) 198:301–6. doi: 10.1016/j.puhe.2021.07.042 34507136

[12] Park Y Kim NH Kwon TY Kim SG. A novel adiposity index as an integrated predictor of cardiometabolic disease morbidity and mortality. Sci Rep (2018) 8(1):16753. doi: 10.1038/s41598-018-35073-4 30425288 PMC6233180

[13] Kim JY Choi J Vella CA Criqui MH Allison MA Kim NH. Associations between weight-adjusted waist index and abdominal fat and muscle mass: multi-ethnic study of atherosclerosis. Diabetes Metab J (2022) 46(5):747–55. doi: 10.4093/dmj.2021.0294 PMC9532169 35350091

[14] Fillo J Levcikova M Ondrusova M Breza J Labas P. Importance of different grades of abdominal obesity on testosterone level, erectile dysfunction, and clinical coincidence. Am J Mens Health (2017) 11(2):240–5. doi: 10.1177/1557988316642213 PMC5675278 27184064

[15] Zhang X Yang B Li N Li H. Prevalence and risk factors for erectile dysfunction in Chinese adult males. J Sex Med (2017) 14(10):1201–8. doi: 10.1016/j.jsxm.2017.08.009 28874333

[16] Curtin LR Mohadjer LK Dohrmann SM Kruszon-Moran D Mirel LB Carroll MD. National health and nutrition examination survey: sample design, 2007-2010. Vital Health Stat 2 (2013) 160):1–23. 25090039

[17] Derby CA Araujo AB Johannes CB Feldman HA McKinlay JB. Measurement of erectile dysfunction in population-based studies: the use of a single question self-assessment in the Massachusetts Male aging study. Int J Impot Res (2000) 12(4):197–204. doi: 10.1038/sj.ijir.3900542 11079360

[18] Farag YMK Guallar E Zhao D Kalyani RR Blaha MJ Feldman DI. Vitamin d deficiency is independently associated with greater prevalence of erectile dysfunction: the national health and nutrition examination survey (Nhanes) 2001-2004. Atherosclerosis (2016) 252:61–7. doi: 10.1016/j.atherosclerosis.2016.07.921 PMC5035618 27505344

[19] Courtney JB Russell MA Conroy DE. Tobacco and cannabis use as moderators of the association between physical activity and alcohol use across the adult lifespan in the united states: nhanes, 2005-2016. Prev Med (2022) 155:106931. doi: 10.1016/j.ypmed.2021.106931 34954238 PMC8886825

[20] DeLong ER DeLong DM Clarke-Pearson DL. Comparing the areas under two or more correlated receiver operating characteristic curves: a nonparametric approach. Biometrics (1988) 44(3):837–45. doi: 10.2307/2531595 3203132

[21] Bacon CG Mittleman MA Kawachi I Giovannucci E Glasser DB Rimm EB. A prospective study of risk factors for erectile dysfunction. J Urol (2006) 176(1):217–21. doi: 10.1016/s0022-5347(06)00589-1 16753404

[22] Riedner CE Rhoden EL Ribeiro EP Fuchs SC. Central obesity is an independent predictor of erectile dysfunction in older men. J Urol (2006) 176(4 Pt 1):1519–23. doi: 10.1016/j.juro.2006.06.049 16952671

[23] Corona G Mannucci E Fisher AD Lotti F Petrone L Balercia G. Low levels of androgens in men with erectile dysfunction and obesity. J Sex Med (2008) 5(10):2454–63. doi: 10.1111/j.1743-6109.2008.00856.x 18494771

[24] Mintziori G Nigdelis MP Mathew H Mousiolis A Goulis DG Mantzoros CS. The effect of excess body fat on female and Male reproduction. Metabolism (2020) 107:154193. doi: 10.1016/j.metabol.2020.154193 32119876

[25] Vignozzi L Filippi S Comeglio P Cellai I Morelli A Marchetta M. Estrogen mediates metabolic syndrome-induced erectile dysfunction: a study in the rabbit. J Sex Med (2014) 11(12):2890–902. doi: 10.1111/jsm.12695 25243860

[26] El Assar M Ruiz de Adana JC Angulo J Pindado Martínez ML Hernández Matías A Rodríguez-Mañas L. Preserved endothelial function in human obesity in the absence of insulin resistance. J Transl Med (2013) 11:263. doi: 10.1186/1479-5876-11-263 24138787 PMC4016214

[27] Silva AB Sousa N Azevedo LF Martins C. Physical activity and exercise for erectile dysfunction: systematic review and meta-analysis. Br J Sports Med (2017) 51(19):1419–24. doi: 10.1136/bjsports-2016-096418 27707739

[28] Morelli A Filippi S Comeglio P Sarchielli E Cellai I Pallecchi M. Physical activity counteracts metabolic syndrome-induced hypogonadotropic hypogonadism and erectile dysfunction in the rabbit. Am J Physiol Endocrinol Metab (2019) 316(3):E519–e35. doi: 10.1152/ajpendo.00377.2018 30645174

[29] Duman DG Biçakci E Çelikel ÇA Akbal C. Nonalcoholic fatty liver disease is associated with erectile dysfunction: a prospective pilot study. J Sex Med (2016) 13(3):383–8. doi: 10.1016/j.jsxm.2015.12.030 26853046

[30] Vignozzi L Filippi S Comeglio P Cellai I Sarchielli E Morelli A. Nonalcoholic steatohepatitis as a novel player in metabolic syndrome-induced erectile dysfunction: an experimental study in the rabbit. Mol Cell Endocrinol (2014) 384(1-2):143–54. doi: 10.1016/j.mce.2014.01.014 24486698

[31] Silva G Lima S Reis BFD Macruz CF Postigo S. Evaluation of obesity influence in the sexual function of postmenopausal women: a cross-sectional study. Rev Bras Ginecol Obstet (2019) 41(11):660–7. doi: 10.1055/s-0039-1700795 PMC10316813 31745959

[32] Werlinger K King TK Clark MM Pera V Wincze JP. Perceived changes in sexual functioning and body image following weight loss in an obese female population: a pilot study. J Sex Marital Ther (1997) 23(1):74–8. doi: 10.1080/00926239708404419 9094038

[33] Ding C Shi Y Li J Li M Hu L Rao J. Association of weight-Adjusted-Waist index with all-cause and cardiovascular mortality in China: a prospective cohort study. Nutr Metab Cardiovasc Dis (2022) 32(5):1210–7. doi: 10.1016/j.numecd.2022.01.033 35277327

[34] Li Q Qie R Qin P Zhang D Guo C Zhou Q. Association of weight-Adjusted-Waist index with incident hypertension: the rural Chinese cohort study. Nutr Metab Cardiovasc Dis (2020) 30(10):1732–41. doi: 10.1016/j.numecd.2020.05.033 32624344

[35] Terentes-Printzios D Ioakeimidis N Rokkas K Vlachopoulos C. Interactions between erectile dysfunction, cardiovascular disease and cardiovascular drugs. Nat Rev Cardiol (2022) 19(1):59–74. doi: 10.1038/s41569-021-00593-6 34331033

[36] Miner M Parish SJ Billups KL Paulos M Sigman M Blaha MJ. Erectile dysfunction and subclinical cardiovascular disease. Sex Med Rev (2019) 7(3):455–63. doi: 10.1016/j.sxmr.2018.01.001 29396281

[37] Mostafaei H Mori K Hajebrahimi S Abufaraj M Karakiewicz PI Shariat SF. Association of erectile dysfunction and cardiovascular disease: an umbrella review of systematic reviews and meta-analyses. BJU Int (2021) 128(1):3–11. doi: 10.1111/bju.15313 33260254 PMC8359379

[38] Maseroli E Scavello I Vignozzi L. Cardiometabolic risk and female sexuality-part i. risk factors and potential pathophysiological underpinnings for female vasculogenic sexual dysfunction syndromes. Sex Med Rev (2018) 6(4):508–24. doi: 10.1016/j.sxmr.2018.02.009 29730315

[39] Maseroli E Fanni E Cipriani S Scavello I Pampaloni F Battaglia C. Cardiometabolic risk and female sexuality: focus on clitoral vascular resistance. J Sex Med (2016) 13(11):1651–61. doi: 10.1016/j.jsxm.2016.09.009 27692844

[40] Kim NH Park Y Kim NH Kim SG. Weight-adjusted waist index reflects fat and muscle mass in the opposite direction in older adults. Age Ageing (2021) 50(3):780–6. doi: 10.1093/ageing/afaa208 33035293

[41] Janiszewski PM Janssen I Ross R. Abdominal obesity and physical inactivity are associated with erectile dysfunction independent of body mass index. J Sex Med (2009) 6(7):1990–8. doi: 10.1111/j.1743-6109.2009.01302.x 19453892

[42] Huang PL. A comprehensive definition for metabolic syndrome. Dis Model Mech (2009) 2(5-6):231–7. doi: 10.1242/dmm.001180 PMC2675814 19407331

[43] Heidler S Temml C Broessner C Mock K Rauchenwald M Madersbacher S. Is the metabolic syndrome an independent risk factor for erectile dysfunction? J Urol (2007) 177(2):651–4. doi: 10.1016/j.juro.2006.09.043 17222651

[44] García-Cruz E Leibar-Tamayo A Romero J Piqueras M Luque P Cardeñosa O. Metabolic syndrome in men with low testosterone levels: relationship with cardiovascular risk factors and comorbidities and with erectile dysfunction. J Sex Med (2013) 10(10):2529–38. doi: 10.1111/jsm.12265 23898860

[45] Romero-Corral A Somers VK Sierra-Johnson J Korenfeld Y Boarin S Korinek J. Normal weight obesity: a risk factor for cardiometabolic dysregulation and cardiovascular mortality. Eur Heart J (2010) 31(6):737–46. doi: 10.1093/eurheartj/ehp487 PMC2838679 19933515

[46] Pizzol D Smith L Fontana L Caruso MG Bertoldo A Demurtas J. Associations between body mass index, waist circumference and erectile dysfunction: a systematic review and meta-analysis. Rev Endocr Metab Disord (2020) 21(4):657–66. doi: 10.1007/s11154-020-09541-0 32002782

[47] Yassin AA Nettleship JE Salman M Almehmadi Y. Waist circumference is superior to weight and bmi in predicting sexual symptoms, voiding symptoms and psychosomatic symptoms in men with hypogonadism and erectile dysfunction. Andrologia (2017) 49(4). doi: 10.1111/and.12634 27400881

[48] Amato MC Giordano C Galia M Criscimanna A Vitabile S Midiri M. Visceral adiposity index: a reliable indicator of visceral fat function associated with cardiometabolic risk. Diabetes Care (2010) 33(4):920–2. doi: 10.2337/dc09-1825 PMC2845052 20067971

[49] Dursun M Besiroglu H Cakir SS Otunctemur A Ozbek E. Increased visceral adiposity index associated with sexual dysfunction in men. Aging Male (2018) 21(3):187–92. doi: 10.1080/13685538.2017.1406468 29166824

[50] Bolat MS Kocamanoglu F Ozbek ML Buyukalpelli R Asci R. Can high visceral adiposity index be a risk factor for sexual dysfunction in sexually active men? J Sex Med (2020) 17(10):1926–33. doi: 10.1016/j.jsxm.2020.06.014 32712095

[51] Antonopoulos AS Tousoulis D. The molecular mechanisms of obesity paradox. Cardiovasc Res (2017) 113(9):1074–86. doi: 10.1093/cvr/cvx106 28549096

